# Isoliquiritigenin Inhibits Gastric Cancer Stemness, Modulates Tumor Microenvironment, and Suppresses Tumor Growth through Glucose-Regulated Protein 78 Downregulation

**DOI:** 10.3390/biomedicines10061350

**Published:** 2022-06-08

**Authors:** Chien-Hsing Lee, Hsin-Yi Tsai, Chun-Lin Chen, Jen-Lung Chen, Chao-Chun Lu, Yi-Ping Fang, Deng-Chyang Wu, Yaw-Bin Huang, Ming-Wei Lin

**Affiliations:** 1 Department of Pharmacology, School of Post-Baccalaureate Medicine, College of Medicine, Kaohsiung Medical University, Kaohsiung 80708, Taiwan; chlee0818@kmu.edu.tw; 2 Drug Development and Value Creation Research Center, Kaohsiung Medical University Hospital, Kaohsiung 80708, Taiwan; 3 Department of Medical Research, Kaohsiung Medical University Hospital, Kaohsiung 80708, Taiwan; 4 Department of Biological Science and Technology, National Pingtung University of Science and Technology, Pingtung 91201, Taiwan; 5 School of Pharmacy, Kaohsiung Medical University, Kaohsiung 80708, Taiwan; y7952pipi@gmail.com (H.-Y.T.); anastasialulululu@gmail.com (C.-C.L.); ypfang@kmu.edu.tw (Y.-P.F.); 6 Department of Medical Research, E-Da Hospital/E-Da Cancer Hospital, Kaohsiung 82445, Taiwan; 7 Department of Biological Science, National Sun Yat-sen University, Kaohsiung 80424, Taiwan; chunlinchen@mail.nsysu.edu.tw; 8 Division of General Surgery, Department of Surgery, E-Da Hospital, Kaohsiung 82445, Taiwan; sardo0926@gmail.com; 9 Division of Gastroenterology, Department of Internal Medicine, Kaohsiung Medical University Hospital, Kaohsiung 80708, Taiwan; dechwu@yahoo.com; 10 Department of Medicine, College of Medicine, Kaohsiung Medical University, Kaohsiung 80708, Taiwan; 11 Regenerative Medicine and Cell Therapy Research Center, Kaohsiung Medical University, Kaohsiung 80708, Taiwan; 12 Department of Nursing, College of Medicine, I-Shou University, Kaohsiung 82445, Taiwan

**Keywords:** GRP78, gastric cancer, cancer stemness, tumor microenvironment, chemosensitivity, isoliquiritigenin

## Abstract

Chemotherapy is the treatment of choice for gastric cancer; however, the currently available therapeutic drugs for treatment have limited efficacy. Cancer stemness and the tumor microenvironment may play crucial roles in tumor growth and chemoresistance. Glucose-regulated protein 78 (GRP78) is an endoplasmic reticulum chaperone facilitating protein folding and cell homeostasis during stress and may participate in chemoresistance. Isoliquiritigenin (ISL) is a bioactive flavonoid found in licorice. In this study, we demonstrated the role of GRP78 in gastric cancer stemness and evaluated GRP78-mediated stemness inhibition, tumor microenvironment regulation, and chemosensitivity promotion by ISL. ISL not only suppressed GRP78-mediated gastric cancer stem cell–like characteristics, stemness-related protein expression, and cancer-associated fibroblast activation but also gastric tumor growth in xenograft animal studies. The findings indicated that ISL is a promising candidate for clinical use in combination chemotherapy.

## 1. Introduction

Gastric cancer is the most common malignancy and the leading cause of cancer deaths worldwide [1]. Numerous studies have reported that chemotherapeutic resistance in solid tumors in the gastrointestinal tract results from genetic heterogeneity in tumor cells. Cancer cells possessing the ability to self-renew and maintain stemness may cause cancer recurrence and contribute to chemoresistance. Suppression of cancer stemness may be a novel target for gastric cancer therapy in precision medicine [2,3].

Glucose-regulated protein 78 (GRP78) is a major chaperone in the endoplasmic reticulum (ER) that regulates many biological functions, including protein folding and cell homeostasis, during the stress-induced unfolded protein response (UPR) [4,5]. Our previous study identified GRP78 as a biomarker for human gastric tumors [6]. Moreover, GRP78 expression was positively related to poor prognosis and served as a marker of response to preoperative chemotherapy in patients with various cancers, including breast cancer, pulmonary adenocarcinoma, myeloma, and pancreatic cancer [7,8,9,10]. Because GRP78 promotes cell survival under stress, GRP78 was reported to maintain cancer stemness in tumor-initiating cells in breast, pancreatic, and head and neck cancers [11,12,13]. Recent studies have suggested that the tumor microenvironment plays a crucial role in cancer stemness and drug resistance to chemotherapy [14]. ER stress and the UPR regulate the tumor microenvironment and affect tumor progression and therapeutic responses [15,16]. However, whether GRP78 plays a role in cancer stemness and chemoresistance in the tumor microenvironment in gastric cancer remains unclear.

Isoliquiritigenin (ISL), a flavonoid derived from licorice, exhibits numerous pharmaceutical properties. For instance, ISL has been demonstrated to attenuate adipose tissue inflammation [17], alleviate diabetic symptoms [18], protect the kidneys during chemotherapy [19], and exhibit anticancer activities [20]. However, mechanisms underlying stemness inhibition and tumor microenvironment regulation in gastric cancer remain to be elucidated.

In this study, we performed the functional and molecular characterization of stemness in human gastric cancer cells and examined the GRP78-mediated inhibition of cancer stemness using ISL by utilizing different functional approaches and stem cell–related markers. We postulated that ISL inhibits gastric cancer stemness markers, regulates the tumor microenvironment, and promotes chemosensitivity through the GRP78-mediated pathway.

## 2. Materials and Methods

### 2.1. Patients Cohort

Twenty GC patients who underwent gastrectomy from 2009 to 2010 at Kaohsiung Medical University Hospital were enrolled. Tumor and normal tissue samples with different stages of gastric cancer were obtained. All the tumor samples and survival data were obtained from Kaohsiung Medical University Hospital. The study protocol was approved by the Ethics Committee on Human Studies of Kaohsiung Medical University Hospital (KMUH-IRB-20120176 and KMUH-IRB-(G11)-20170028). Table 1 summarizes the data regarding the samples.

### 2.2. Cell Culture and Reagent

The human gastric cancer cell line, MKN45, was purchased from DSMZ (ACC-409, DSMZ, Braunschweig, Germany) and cultured in RPMI 1640 medium (Gibco, Waltham, MA, USA) containing 10% fetal bovine serum (FBS; Gibco, Waltham, MA, USA) under 5% CO_2_ at 37 °C. The MKN45 cells were cultured in RPMI 1640 medium (Gibco, Waltham, MA, USA) containing 10% fetal bovine serum (Gibco, Waltham, MA, USA) under 5% CO_2_ at 37 °C. The cells were treated with trypLE reagent (Gibco, Waltham, MA, USA). MKN45/ctrl (shLacZ, clone ID: TRCN231722), MKN45/GRP78^+^ (GRP78-Bip-pLAS2w cloning vector), and MKN45/sh-GRP78 (shHSPA5, clone ID: TRCN218611) were purchased from the National RNAi Core Facility (RNA technology platform and gene manipulation core, Academia Sinica, Taipei City, Taiwan). The transfected MKN45 cells were cultured in RPMI 1640 medium (Gibco, Waltham, MA, USA) containing 10% FBS and 3 mg/mL puromycin under 5% CO_2_ at 37 °C. Stock solutions of ISL (Sigma-Aldrich, St. Louis, MO, USA) and 5-fluorouracil (5-FU; Sigma-Aldrich, St. Louis, MO, USA) in dimethyl sulfoxide (DMSO) (Sigma-Aldrich, St. Louis, MO, USA) were prepared and dissolved in culture medium before treatment. The human primary cancer-associated fibroblast h-GCA-N3 cells (a gift from assistant professor Dr. Ming-Hong Lin, Department of Immunology, Kaohsiung Medical University, Taiwan) were cultured in K-medium (Gibco, Waltham, MA, USA) supplemented with 10% FBS (Gibco, Waltham, MA, USA), N-acetyl-L-cysteine (360 μg/mL), and L-ascorbic acid 2-phosphate (51.2 μg/mL) under 5% CO_2_ at 37 °C.

### 2.3. Cell Viability Analysis

The MKN45/ctrl and MKN45/sh-GRP78 cells were seeded in 96-well plates in quadruplicate at 6000 cells/well and cultured for 24 h before treatment. Cell viability was analyzed using the Cell Counting Kit-8 (Sigma-Aldrich, St. Louis, MO, USA) according to the manufacturer’s instructions, and absorbance was measured at 450 nm using a microplate reader.

### 2.4. Western Blot

The tissues or human gastric cancer cells were washed with phosphate-buffered saline (PBS). Total protein samples were extracted, and protein concentrations were measured using the Bio-Rad Bradford Protein Assay (Bio-Rad, Hercules, CA, USA). Equal quantities of total proteins were separated through BOLT BISTRIS PLUS 4–12% sodium dodecyl sulfate–polyacrylamide gel electrophoresis (Thermo Scientific, New York, NY, USA) and transferred onto polyvinylidene fluoride membranes. The membranes were blocked with a blocking buffer (Bio-Rad, Hercules, CA, USA) for 30 min at room temperature and incubated with the primary antibodies SOX2 (1:1000; #23064; Cell Signaling, Danvers, MA, USA), GRP78 (1:1000; #3183; Cell Signaling, Danvers, MA, USA), β-actin (1:5000; #4967; Cell Signaling, Danvers, MA, USA), CREB3L1/OASIS (1:1000; ab137565, Abcam, Cambridge, UK), matrix metallopeptidase 9 (MMP-9; 1:1000; ab283575, Abcam, Cambridge, UK), and α-smooth muscle actin (1:1000; #14968; Cell Signaling, Danvers, MA, USA) at 4 °C after the membranes were washed with PBS with Tween 20. The membranes were incubated with secondary antibodies at room temperature for 1 h and then analyzed using an electrochemiluminescence detection system.

### 2.5. Flow Cytometry

The MKN45, MKN45/ctrl, MKN45/GRP78^+^, and MKN45/sh-GRP78 cells were seeded in six-well plates in quadruplicate at 1 × 10^5^ cells/well for 24 h with or without treatment, washed with cold PBS, and stained with a surface marker antibody for 45 min. After staining, the cells were washed twice with cold PBS before analysis. The expression of cancer stem cell (CSC) markers (CD24, CD44, and LGR5; BD Biosciences, San Jose, CA, USA) on the human gastric cancer cells was analyzed through flow cytometry.

For aldehyde dehydrogenase 1 (ALDH1) analysis, the MKN45 cells were stained using the AldeRed ALDH Detection Assay kit (Sigma-Aldrich, St. Louis, MO, USA) and analyzed through flow cytometry.

### 2.6. Sphere Formation Analysis

For the sphere formation assay, the MKN45, MKN45/GRP78^+^, MKN45/ctrl, or MKN45/sh-GRP78 cells were seeded in low-adhesion culture dishes in serum-free RPMI 1640 medium (Gibco, Waltham, MA, USA) containing 2% B27 supplement (Gibco, Waltham, MA, USA), 20 ng/mL epidermal growth factor (Gibco, Waltham, MA, USA), and 20 ng/mL fibroblast growth factor (Gibco, Waltham, MA, USA) for 7 days. Sphere formation in the cells was evaluated using Image J software.

### 2.7. Soft Agar Colony Formation Analysis

For the colony formation assay, the MKN45, MKN45/GRP78^+^, MKN45/ctrl, or MKN45/sh-GRP78 cells were seeded in six-well dishes in quadruplicate at 2.5 × 10^4^ cells/well and coated in 0.3% agar. After 2 weeks, the colonies were stained with 0.005% crystal violet and evaluated using Image J software.

### 2.8. Measurement of Tumor Growth Factor-β1 Level

The condition medium was collected from the MKN45 or MKN45/ctrl, MKN45/GRP78^+^ or MKN45/sh-GRP78, or h-GCA-N3 cells treated with ISL. The intracellular tumor growth factor (TGF)-β1 level was evaluated using the human TGF-β1 enzyme-linked immunoassay (ELISA) kit (ab108912, Abcam, Cambridge, UK) according to the manufacturer’s instructions.

### 2.9. Measurement of Interleukin-6 Level

The condition medium was collected from the h-GCA-N3 cells treated with ISL. The intracellular interleukin (IL)-6 level was evaluated using the human IL-6 ELISA kit (ab178013, Abcam, Cambridge, UK) according to the manufacturer’s instructions.

### 2.10. Xenograft Tumor Experiments

For GRP78 knockdown experiments, mice were randomly divided into two groups: control (Ctrl; *n* = 4) and GRP78 knockdown (sh-GRP78; *n* = 7). The MKN45 cells stably transfected with GRP78 shRNA or scrambled shRNA were harvested and subcutaneously inoculated (5 × 10^6^ cells/0.1 mL in PBS) into 6-week-old BALB/c nude mice (BioLASCO, Taipei City, Taiwan). The tumor volumes were measured every 2 days and determined using the formula V = L × W2/2 (L, length; W, width). The tumors were harvested after 14 days. For ISL treatment experiments, the mice were randomly divided into four groups: control (Ctrl; *n* = 7), ISL treatment (ISL; *n* = 7), 5-Fu treatment (5-FU; *n* = 7), and combined treatment (ISL + 5-Fu; *n* = 7). For ISL treatment, the MKN45 cells were pretreated with 25 μg/mL ISL for 72 h before injection. The cells were harvested and subcutaneously inoculated (5 × 10^6^ cells/0.1 mL in PBS) into the 6-week-old BALB/c nude mice (BioLASCO Taipei City, Taiwan). For 5-Fu treatment, one week after the subcutaneous injection of cancer cells, the mice were intraperitoneally administered saline or 5-Fu (10 mg/kg) every 2 days for 1 week. The tumor volume was calculated using the formula V = L × W2/2 (L, length; W, width). The tumors were harvested after 14 days

### 2.11. Immunohistochemistry and Hematoxylin plus Eosin Staining

The tissues were embedded in paraffin wax after cutting and dehydrating them with serial alcohol solutions. The paraffin-embedded tissues were cut into 3-μm sections and placed on slides, followed by staining with the GRP78 antibody (1:1000; #3183; Cell Signaling), ki-67 (1:500; SP6; Spring Bioscience, Pleasanton, CA, USA), and α-SMA (1:100; SP171; Spring Bioscience, Pleasanton, CA, USA) for immunohistochemical (IHC) analysis according to the manufacturer’s instructions. For H&E staining, the paraffin embed slides were dewaxed, gradually hydrated through graded alcohol, and were stained in hematoxylin solution and differentiated in 1% hydrochloric alcohol. After rinsing with distilled water, the sides were dehydrated in 95% ethanol, counterstained in 1% eosin solution, washed with 70% ethanol, absolute ethanol, and then were cleared in 2 changes of xylene.

### 2.12. Statistical Analysis

All data were analyzed using GraphPad Prism version 8. All graphs in figures present the mean ± standard deviation (SD). Statistical analysis was performed using Student’s *t* test to compare data between the groups. A *p* value of <0.05 indicated statistical significance. Statistical results are labeled in each figure as * *p* < 0.05, ** *p* < 0.01, or *** *p* < 0.001. Survival analyses were conducted considering the time from diagnosis to the date of the event (death or the last follow-up). Overall and disease-free survival were estimated using the Kaplan–Meier method.

## 3. Results

### 3.1. GRP78 Overexpression in Human Gastric Tumor Cells

**Figure 1:**
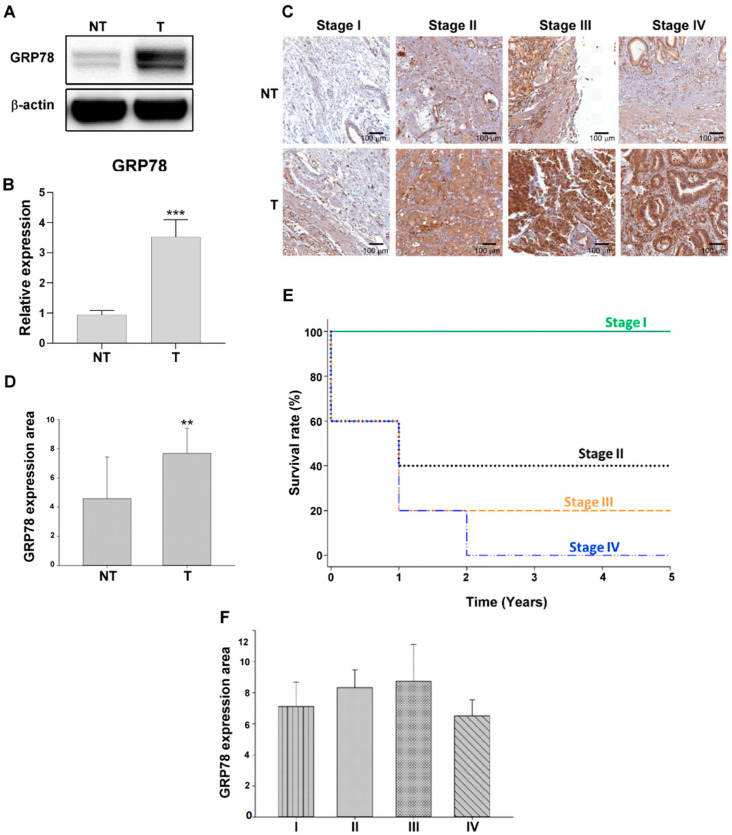
Glucose-regulated protein 78 (GRP78) was overexpressed in patients with gastric cancer. (**A**) Expression of GRP78 in tumor and normal tissues in patients with stage I cancer. (**B**) Quantification of GRP78 expression in the tissue samples of patients with gastric cancer. (**C**) GRP78 expression was analyzed by performing immunohistochemistry (IHC) analysis in tumor and normal tissue samples of patients with stage I to stage IV cancer. (**D**) Quantification of GRP78 expression in the tumor tissue samples of patients with stage I gastric cancer compared with their corresponding normal tissues through IHC staining. (**E**) Kaplan–Meier curves for overall survival rates associated with GRP78 expression in gastric cancer. (**F**) Quantification of GRP78 expression analyzed through IHC staining in the tumor tissues of patients with stage I to stage IV cancer. Data are expressed as the mean ± standard error of mean; *n* ≥ 3 independent experiments, two-tailed Student’s *t* test: ** *p* < 0.01, *** *p* < 0.005.

Our previous proteomics study identified GRP78 as a tumor marker in gastric cancer [6]. In this study, we examined GRP78 expression in the clinical tumor and normal tissue samples of the patients with different stages of gastric cancer through Western blot and IHC analyses. GRP78 expression was significantly upregulated by over 1.5-fold (*p* < 0.01) in stage I gastric tumor tissue samples compared with their corresponding normal tissue samples (Figure 1A,B). The results of IHC analysis (Figure 1C) indicated that GRP78 expression was upregulated from stage I to stage IV tumor tissue samples compared with their corresponding normal tissue samples, and GRP78 expression was associated with decreased overall survival in the patients with gastric cancer (Figure 1E). GRP78 expression was slightly downregulated in stage IV tumor tissue samples compared with stage III tumor tissue samples; however, the difference was not significant (Figure 1F).

### 3.2. GRP78 Overexpression in Stem Cell-like Spheroid-Forming Human Gastric Cancer Cells

**Figure 2:**
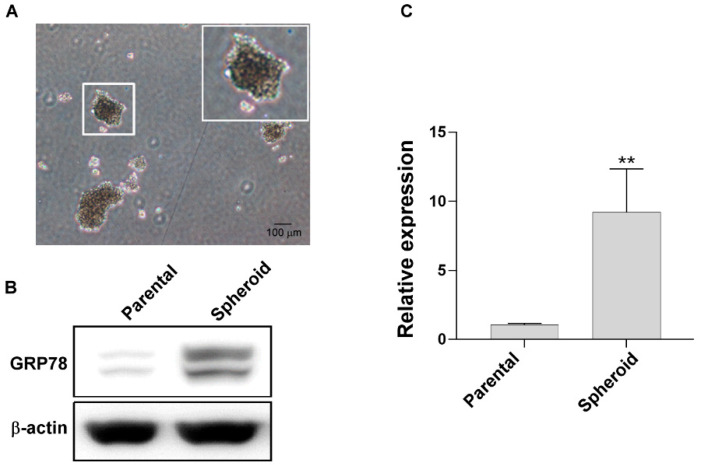
Glucose-regulated protein 78 (GRP78) was overexpressed in spheroid gastric cancer cells. (**A**) A representative picture demonstrating the MKN45 cells with the spheroid-forming ability. (**B**) Protein expression of GRP78 in parental and spheroid body-forming MKN45 cells. (**C**) Quantification of GRP78 expression in parental and spheroid body–forming MKN45 cells. Data are expressed as the mean ± standard error of mean; *n* ≥ 3 independent experiments, two-tailed Student’s *t* test: ** *p* < 0.01.

The spheroid-forming MKN45 cells (Figure 2A) were harvested using low-adhesion culture plates under serum-free culture conditions. GRP78 was overexpressed in the spheroid-forming cells compared with the normal cells (Figure 2B,C), indicating that the stem cell–like gastric cancer cells expressed a higher GRP78 protein level.

### 3.3. GRP78 Overexpression in Human Gastric Cancer Cells Promote Stem Cell-like Characteristics 

**Figure 3:**
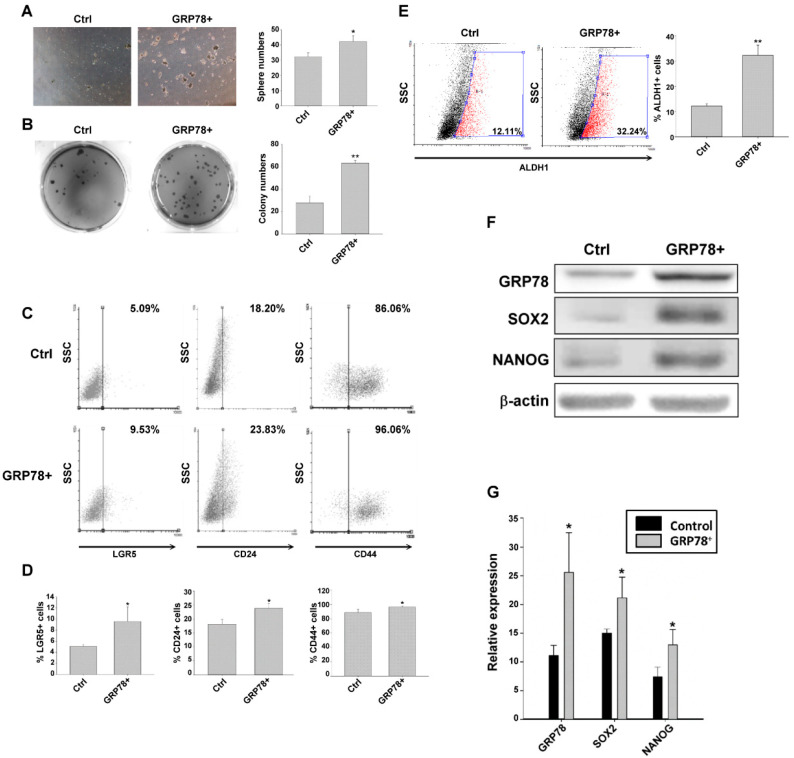
Glucose-regulated protein 78 (GRP78) promoted stemness in MKN45 cells. (**A**) Representative pictures showing spheroid body-forming and (**B**) colony-forming MKN45/ctrl and MKN45/GRP78^+^ cells. (**C**) Stemness-related surface makers (LGR5, CD24, and CD44) were analyzed through flow cytometry in MKN45/ctrl and MKN45/GRP78^+^ cells. (**D**) Quantification of the expression of surface makers (LGR5, CD24, and CD44). (**E**) Aldehyde dehydrogenase 1-positive cells were analyzed through flow cytometry among MKN45/ctrl and MKN45/GRP78^+^ cells. (**F**) Protein expression of stemness-related transcription factors (SOX2 and Nanog) and GRP78 in MKN45/ctrl and MKN45/GRP78^+^ cells. (**G**) Quantification of SOX2, Nanog, and GRP78 expression in MKN45/ctrl and MKN45/GRP78^+^ cells. Data are expressed as the mean ± standard error of mean; *n* ≥ 3 independent experiments, two-tailed Student’s *t* test: * *p* < 0.05, ** *p* < 0.01.

To determine whether GRP78 upregulation promotes gastric cancer cell stemness, we examined the stem cell–like characteristics of the GRP78-overexpressing MKN45 cells (GRP78^+^) compared with the normal MKN45 cells. As shown in Figure 3A,B, the GRP78^+^ MKN45 cells promoted cell spheroid formation and colony formation. The expression of gastric cancer stemness–related surface markers was increased in the GRP78^+^ MKN45 cells (Figure 3C,D). Moreover, the stemness-related transcriptional factors SOX2 and Nanog were upregulated in the GRP78-expressing MKN45 cells (Figure 3F,G). The results suggested that GRP78 promotes gastric cancer stem cell–like characteristics.

### 3.4. ISL Inhibited GRP78 Expression and Suppressed Stem Cell-like Characteristics in Human Gastric Cancer Cells

**Figure 4:**
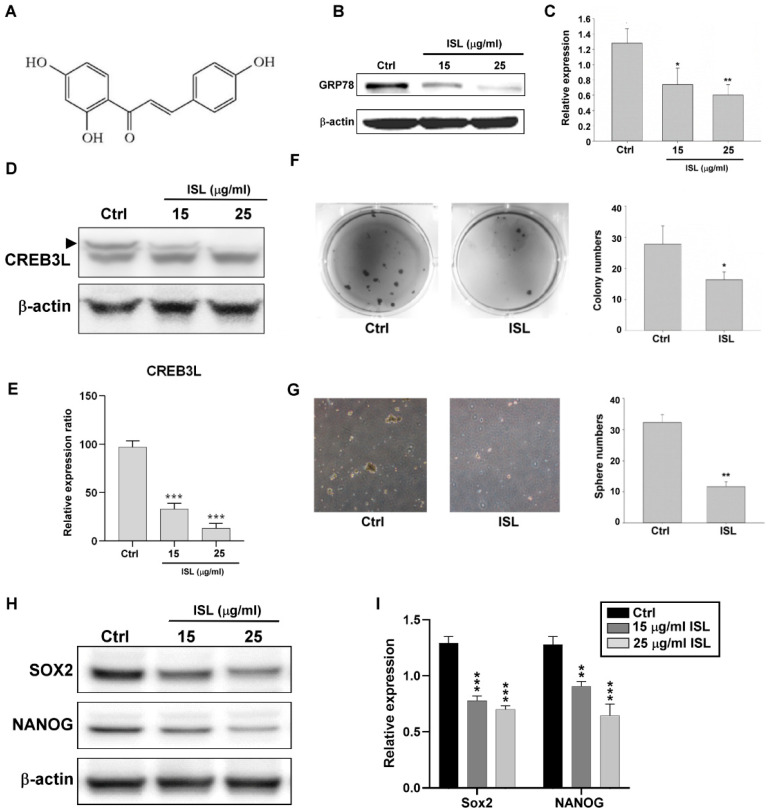
The stemness characteristic was reduced by isoliquiritigenin (ISL) treatment. (**A**) Structural formula of ISL. (**B**) Glucose-regulated protein 78 (GRP78) expression in MKN45 cells was inhibited after treatment with 15 or 25 μg/mL ISL for 72 h. (**C**) Quantification of GRP78 expression after ISL treatment. (**D**) The protein expression of the transcription factor CREB3L1 was downregulated in MKN45 cells after treatment with 15 or 25 μg/mL ISL for 72 h. (**E**) Quantification of CREB3L1. (**F**) Representative pictures demonstrating that the colony-forming MKN45 cells were decreased after treatment with 25 μg/mL ISL. (**G**) Representative pictures demonstrating that the spheroid body-forming MKN45 cells were decreased after treatment with 25 μg/mL ISL. (**H**,**I**) The protein expression of stemness-related transcription factors (SOX2 and Nanog) was downregulated in MKN45 cells after treatment with 15 or 25 μg/mL ISL for 72 h. Data are presented as the mean ± standard error of mean; *n* ≥ 3 independent experiments, two-tailed Student’s *t* test: * *p* < 0.05, ** *p* < 0.01, *** *p* < 0.005.

ISL (Figure 4A) is a natural flavonoid. To evaluate whether ISL suppresses gastric cancer stem cell–like characteristics through GRP78 inhibition, the MKN45 cells were treated with ISL (15 or 25 μg/mL) for 72 h. The results revealed that ISL inhibited GRP78 expression in the MKN45 cells in a dose–dependent manner (Figure 4B,C). CREB3L is a member of the UPR that acts on cyclic adenosine monophosphate (AMP) to promote the expression of target genes including GRP78 [21]. ISL inhibited CREB3L in a dose–dependent manner (Figure 4D,E). Moreover, ISL not only suppressed the formation of colonies and spheroids but also inhibited the expression of the stemness-related transcriptional factors SOX2 and Nanog (Figure 4F–I). ISL may suppress gastric cancer stem cell–like characteristics through CREB3L-mediated GRP78 downregulation.

### 3.5. Knockdown of GRP78 Suppressed Stem Cell-like Characteristics in Human Gastric Cancer Cells

**Figure 5:**
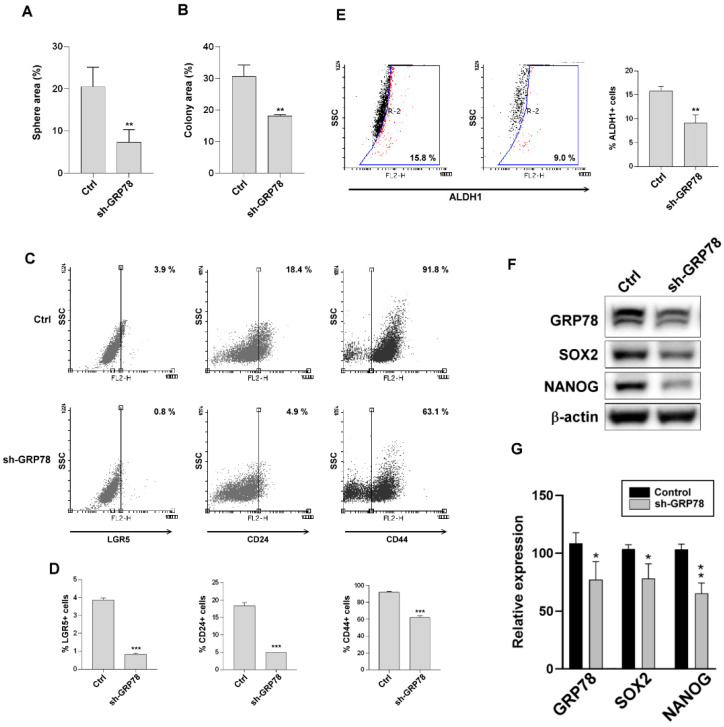
Glucose-regulated protein 78 (GRP78) silencing reduced the stemness capacity of MKN45 cells. (**A**) Representative pictures showing the spheroid-forming capacity between MKN45/ctrl and MKN45/sh-GRP78 cells. (**B**) Representative pictures showing the colony-forming capacity between MKN45/ctrl and MKN45/sh-GRP78 cells. (**C**) Expression of surface makers (LGR5, CD24, and CD44) was analyzed through flow cytometry in MKN45/ctrl and MKN45/sh-GRP78 cells. (**D**) Quantification of surface makers. (**E**) Aldehyde dehydrogenase 1-positive cells were analyzed through flow cytometry in MKN45/ctrl and MKN45/sh-GRP78 cells. (**F**) Protein expression of stemness-related transcription factors (SOX2 and Nanog) and GRP78 was analyzed through Western blot in MKN45/ctrl and MKN45/sh-GRP78 cells. (**G**) Quantification of the expression of stemness markers (SOX2 and Nano) and GRP78. Data are presented as the mean ± standard error of the mean; *n* ≥ 3 independent experiments, two-tailed Student’s *t* test: * *p* < 0.05, ** *p* < 0.01, *** *p* < 0.005.

To confirm whether GRP78 downregulation suppresses gastric cancer cell stemness, the MKN45 (sh-GRP78) cells with GRP78 knockdown were used to evaluate stem cell–like characteristics compared with those of the normal MKN45 cells. As depicted in Figure 5A–G, the MKN45 cells with GRP78 knockdown exhibited decreased formation of colonies and spheroids and decreased expression of gastric cancer stem cell–like markers (LGR5, CD24, CD44, and ALDH1) and stemness-related transcriptional factors (SOX2 and Nanog). The results suggested that GRP78 silencing reduced the stemness capacity in the MKN45 cells. 

### 3.6. Knockdown of GRP78 Inhibited Tumor Growth in Xenograft Tumor Mice

**Figure 6:**
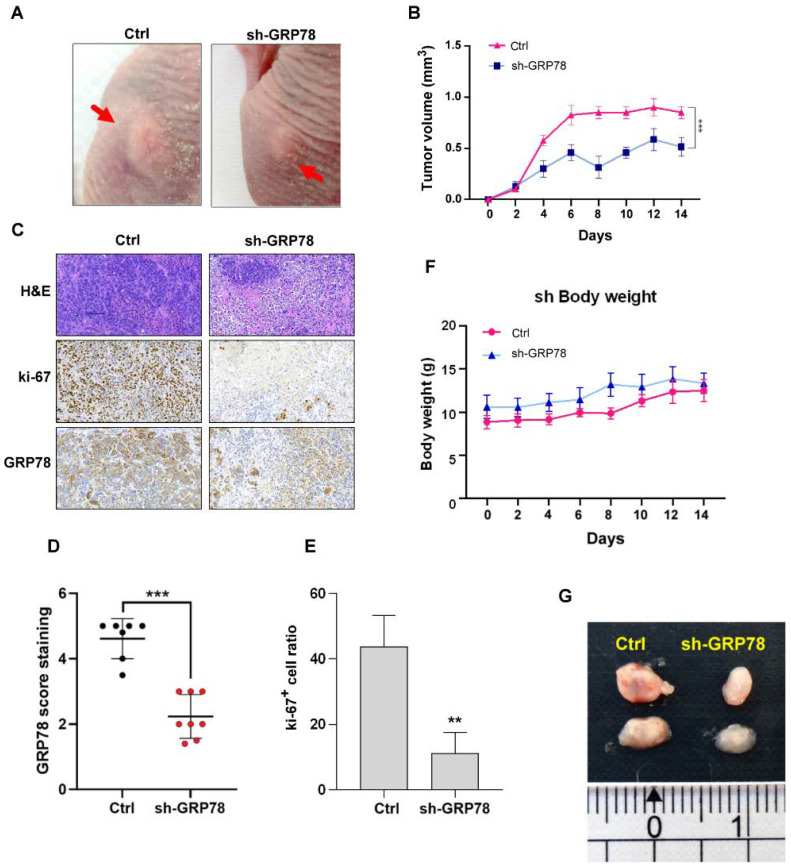
Knockdown of glucose-regulated protein 78 (GRP78) reduced tumor growth in gastric cancer xenografts. (**A**) Schematic of gastric cancer xenografts between MKN45/ctrl and MKN45/shGRP78 groups. (**B**) The curves of tumor growth in mice between MKN45/ctrl and MKN45/shGRP78 groups. (**C**) Representative images of hematoxylin and eosin and immunohistochemical (IHC) staining of ki-67 between MKN45/ctrl and MKN45/shGRP78 groups. (**D**) Representative IHC analysis of GRP78 in MKN45/ctrl and MKN45/shGRP78 gastric cancer xenografts and relative quantification per intensity of staining scoring. (**E**) Representative IHC analysis of ki-67 staining in MKN45/ctrl and MKN45/shGRP78 gastric cancer xenografts and relative quantification per intensity of staining scoring. (**F**) Curves of body weight of mice between MKN45/ctrl and MKN45/shGRP78 groups. (**G**) Tumor sizes of each group. Data are presented as the mean ± standard error of mean; *n* ≥ 4 independent experiments, two-tailed Student’s *t* test: ** *p* < 0.01, *** *p* < 0.005.

To confirm whether GRP78 downregulation inhibited tumor growth in vivo, the nude mice were inoculated with the MKN45 (sh-GRP78) cells with GRP78 knockdown. The results demonstrated that tumor growth was inhibited in the sh-GRP78 group in the xenograft tumor mice (Figure 6A,B,G). The results of IHC analysis indicated that GRP78 and ki-67, a tumor cell proliferation marker, were downregulated in the tumor tissue of the sh-GRP78 groups in Figure 6C–E. 

### 3.7. ISL Regulated the Tumor Microenvironment through GRP78-Mediated TGF-β1 Expression

**Figure 7:**
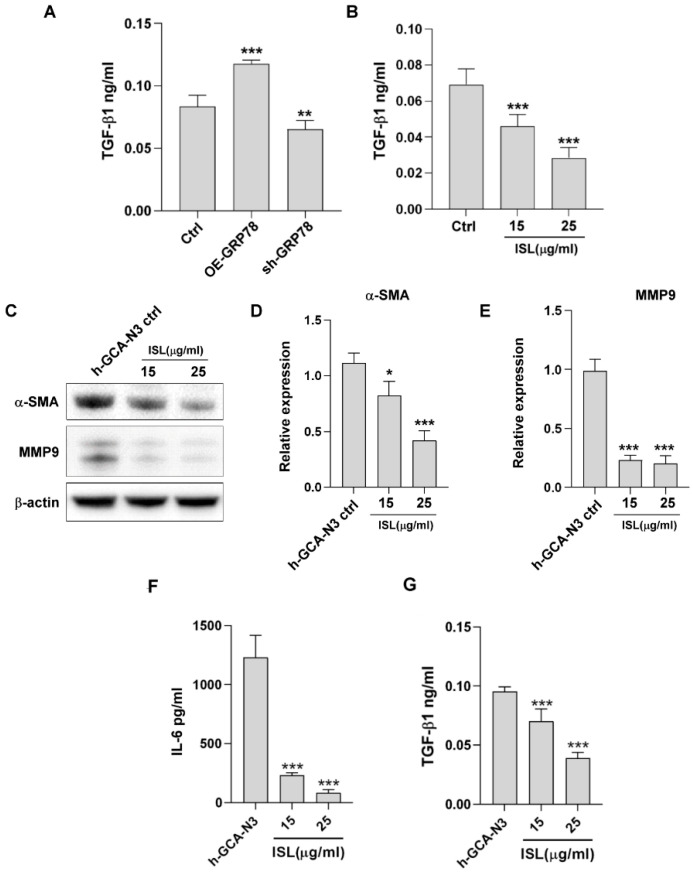
Glucose-regulated protein 78 (GRP78) expression in gastric cancer cells induces cancer-associated fibroblast activity. (**A**) Levels of tumor growth factor (TGF)-β1 in the condition medium of MKN45/ctrl, MKN45/GRP78^+^, and MKN45/sh-GRP78 cells. (**B**) Levels of TGF-β1 in the condition medium in MKN45 cells with or without ISL treatment. (**C**) Protein expression of α-SMA and matrix metalloproteinase (MMP-9) was analyzed through a Western blot after treatment with 15 and 25 μg/mL isoliquiritigenin (ISL) for 48 h in h-GCA N3 cells. (**D**,**E**) Quantification of α-SMA and MMP-9 expression was analyzed through a Western blot after treatment with 15 and 25 μg/mL ISL for 48 h. (**F**) Levels of IL-6 in the conditioned medium in h-GCA N3 cells with or without ISL treatment. (**G**) Levels of TGF-β1 in the conditioned medium in h-GCA N3 cells with or without ISL treatment. Data are presented as the mean ± standard error of mean; *n* ≥ 3 independent experiments, two-tailed Student’s *t* test: * *p* < 0.05, ** *p* < 0.01, *** *p* < 0.005.

TGF-β1 is a multifunctional cytokine involved in forming the tumor microenvironment, activating cancer-associated fibroblasts (CAFs), and eventually promoting tumor metastasis. TGF-β1 activates CAFs, and CAFs mediate cancer stemness through extracellular matrix remodeling [22,23]. CAFs secrete MMP-9 to promote epithelial–mesenchymal transition (EMT) and stemness genes [24,25]. To determine whether GRP78 or ISL regulates TGF-β1 in gastric cancer cells and suppresses CAF activation, TGF-β1 secreted from the MKN45 or primary CAF h-GCA-N3 cells in the condition medium was evaluated using ELISA. The results demonstrated that GRP78 regulated TGF-β1 secretion in the MKN45 cells, and the inhibition of GRP78 by ISL suppressed TGF-β1 secretion (Figure 7A,B). CAF activation was evaluated by examining α-SMA or MMP-9 expression. ISL inhibited α-SMA and MMP-9 expression in the h-GCA-N3 cells in ISL-treated MKN45 condition medium (Figure 7C–E). Moreover, ISL inhibited IL-6 or TGF-β1 secretion from the h-GCA-N3 cells (Figure 7F,G). 

### 3.8. ISL Inhibited 5-FU-Induced GRP78-Mediated Gastric Cancer Stemness

**Figure 8:**
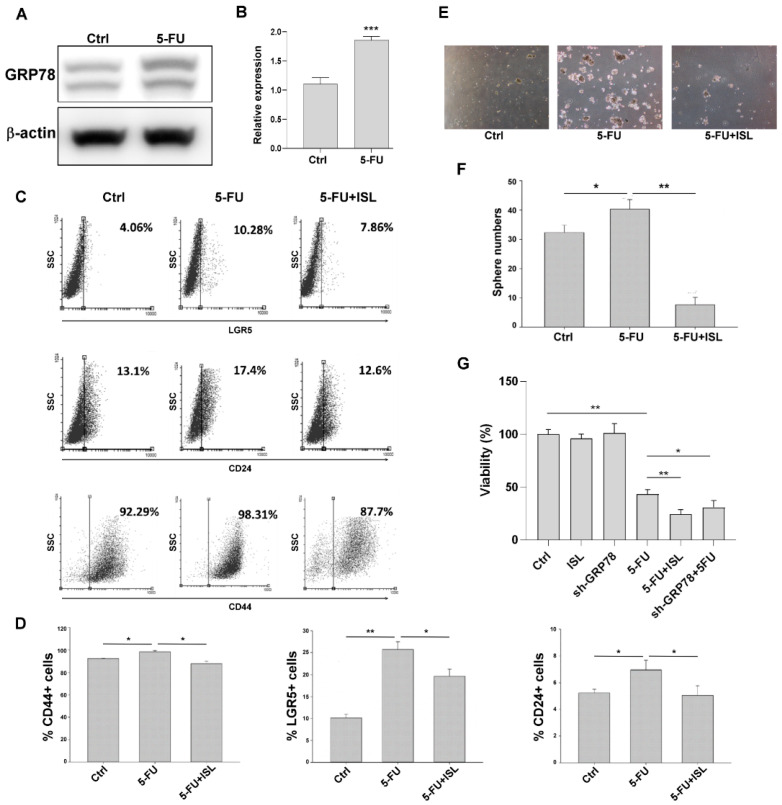
Isoliquiritigenin (ISL) inhibited 5-fluorouracil (5-FU)-induced glucose-regulated protein 78 (GRP78)-mediated stemness. (**A**) GRP78 expression was analyzed through Western blot after treatment with 0.5 μM 5-FU for 72 h. (**B**) Quantification of GRP78 expression. (**C**) Expression of surface markers (LGR5, CD24, and CD44) was analyzed through flow cytometry in MKN45 cells after treatment with ISL or ISL combined with 5-FU. (**D**) Quantification of surface makers. (**E**,**F**) Representative pictures showing the spheroid-forming capacity of MKN45 cells treated with or without ISL combined with 5-FU. (**G**) The viability of the MKN45, ISL-treated MKN45, MKN45/sh-GRP78, 5-FU-treated MKN45, 5-FU+ISL-treated MKN45, and 5-Fu-treated MKN45/sh-GRP78 cells was evaluated. Data are presented as the mean ± standard error of mean; *n* ≥ 3 independent experiments, two-tailed Student’s *t* test: * *p* < 0.05, ** *p* < 0.01, *** *p* < 0.005.

Cells of five-FU were reported to induce ER stress and modulate GRP78 expression in cancer cells, thus possibly resulting in chemoresistance [26,27]. Our results revealed that 5-FU induced GRP78 expression in the MKN45 cells (Figure 8A,B). To determine whether the inhibition of GRP78 by ISL suppresses 5-FU-mediated cancer stemness in human gastric cancer cells, we evaluated the expression of the stemness surface markers and spheroid formation capacity. The results indicated that ISL suppressed the expression of 5-FU-induced stemness-related surface markers (LGR5, CD24, and CD44) and the spheroid formation capacity (Figure 8C–F). Knockdown of GRP78 or ISL treatment promoted chemosensitivity to 5-FU (Figure 8G).

### 3.9. ISL Enhanced Chemosensitivity to 5-FU and Inhibited Tumor Growth in Xenograft Tumor Mice

**Figure 9:**
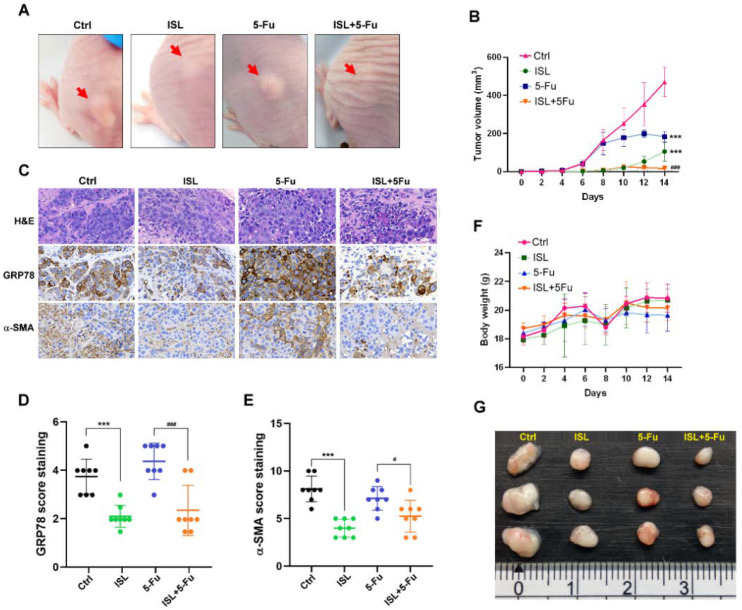
Pretreatment of isoliquiritigenin (ISL) reduced tumor growth and promoted chemosensitivity to 5-fluorouracil (5-FU). (**A**) Schematic of gastric cancer xenografts in each group. (**B**) Tumor growth curves of the Ctrl, ISL, 5-FU, and ISL+5-FU groups. (**C**) Representative images of hematoxylin and eosin and immunohistochemical (IHC) staining of GRP78 and α-SMA in each group. (**D**) Representative IHC analysis of GRP78 in gastric tumor xenografts (**E**) Representative IHC analysis of α-SMA in gastric tumor xenografts. (**F**) Curves of body weight of mice in each group. (**G**) Gastric tumors harvested from each group. Data are presented as the mean ± standard error of mean; *n* ≥ 7 independent experiments, two-tailed Student’s *t* test: ^#^ *p* < 0.05, ^###^ *p* < 0.005, *** *p* < 0.005.

To confirm whether ISL-induced GRP78 downregulation inhibited tumor growth in vivo, the MKN45 cells were pretreated with 25 μg/mL ISL for 72 h before inoculation in the nude mice. The results demonstrated that tumor growth was inhibited in the ISL-treated group in the xenograft tumor mice (Figure 9A,B,G). Moreover, GRP78 was upregulated in the 5-FU-treated tumor, and ISL inhibited GRP78 and α-SMA expression in the 5-FU-treated groups in IHC analysis (Figure 9C–E). The findings of the xenograft tumor study indicated that ISL inhibited tumor growth and promoted chemosensitivity (Figure 9B,G).

### 3.10. ISL Suppresses Cancer Stemness–Mediated Chemoresistance, Tumor Microenvironment, and Tumor Growth by GRP78 Inhibition

**Figure 10:**
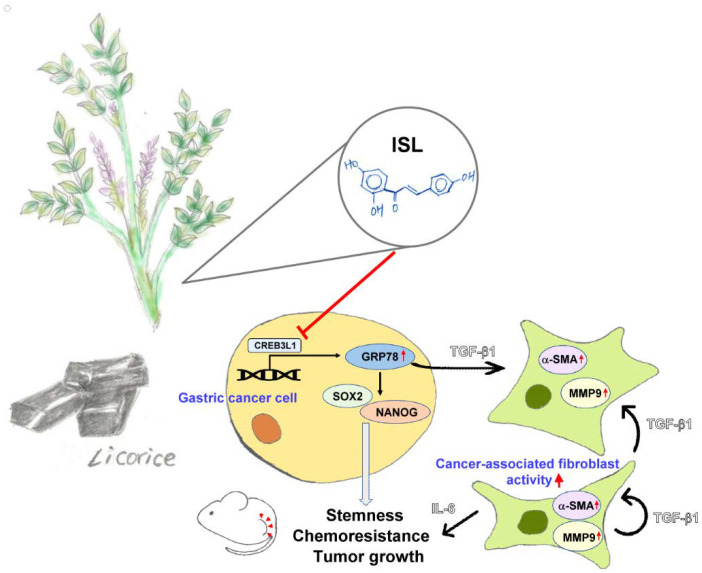
Graphical scheme depicting that isoliquiritigenin (ISL) inhibited gastric cancer stemness and tumor growth and regulated the tumor microenvironment by downregulating GRP78.

The results of our study indicated that ISL regulated the tumor microenvironment and inhibited GRP78 expression by downregulating the transcriptional factor CREB3L1 and suppressed stem cell–like characteristics in human gastric cancer cells. Furthermore, downregulation of GRP78 by ISL inhibited TGF-β1 secretion from the cancer cells and prevented the activation of CAFs and the inhibition of IL-6, TGF-β1, and MMP-9 by CAFs (Figure 10).

## 4. Discussion

The results of this study demonstrated that GRP78 plays a crucial role in gastric cancer stemness and that ISL inhibited gastric CSC markers through the GRP78-mediated pathway, regulated the tumor microenvironment, and enhanced chemosensitivity to 5-FU. ISL regulates GRP78 expression in different cancer cells by different mechanisms [28,29]. CREB3L1 is a member of the UPR and acts on cyclic AMP to promote the expression of target genes including GRP78 [21]. The inhibition of GRP78 expression in human gastric cancer cells by ISL is mediated by its transcriptional factor, CREB3L1. 

GRP78 upregulation in human gastric tumor tissues was confirmed in our clinical study. GRP78 is an ER chaperone facilitating protein folding and cell homeostasis during the ER stress-induced UPR [4,5]. The UPR is an adaptive mechanism that regulates protein and cellular homeostasis. In addition, the UPR plays a crucial role in cancer and contributes to resistance to chemotherapeutics. Increasing evidence indicates the involvement of the UPR in oncogenic reprogramming and the regulation of tumor cells with stem cell properties. Mechanisms through which UPR branches regulate stemness in cancer should be elucidated [30,31]. Recent studies have highlighted the importance of GRP78-mediated cancer stemness [11,32]. 

Gastric cancer stemness can be determined by investigating the expression of cell surface markers, namely CD24, CD44, and LGR5, and stemness-related transcriptional factors, namely Nanog and SOX2. Nanog is widely expressed in human cancer and is involved in self-renewal, metastasis, and chemoresistance [33,34]. SOX2 is involved in the maintenance of an undifferentiated cellular phenotype and often leads to increased chemotherapy resistance in cancer [35,36]. Another functional marker, ALDH1, which is a detoxifying enzyme responsible for oxidation, is widely used to characterize cancer stemness [37] and serves as an indicator for poor prognosis in gastric cancer [38,39].

Knockdown of GRP78 expression or inhibition of GRP78 by ISL downregulated CD24, CD44, LGR5, SOX2, and Nanog in gastric cancer in our study. Similarly, other studies have indicated that ER stress and UPR activation regulate glioblastoma stemness through SOX2 modulation [40]. Inhibition of GRP78 by antibodies effectively reduced the cell surface expression of CD44 and the invasiveness of tamoxifen-resistant breast cancer cells [41]. Suppression of GRP78 downregulated CD24 expression in colorectal cancer and increased sensitivity to the chemotherapy agent oxaliplatin [42]. Moreover, ISL inhibited GRP78 in oral cancer cells. ISL not only inhibited the self-renewal ability but also reduced the expression of cancer stemness markers, including ALDH1 and CD44, in the GRP78-mediated pathway [29].

TGF-β1 was inhibited by ISL or GRP78 knockdown gastric cells in our study. CAFs in the tumor microenvironment might sustain the stemness of gastric cancer cells through TGF-β signaling [43]. TGF-β1 is a multifunctional cytokine and increases the α-SMA expression level of CAFs and promotes EMT, thereby enhancing stemness and chemoresistance in tumor cells [44]. Through paracrine signaling, TGF-β1 can help in the formation of the tumor microenvironment by activating CAFs to produce the extracellular matrix and IL-6. IL-6 secreted by CAFs promotes cancer stemness, chemoresistance, and invasion through Nanog activation and eventually promotes tumor growth and metastasis [45,46,47]. Our results indicated that ISL inhibited TGF-β1 through GRP78-mediated pathways in gastric cancer cells. The inhibition of TGF-β1 secreted by gastric cancer cells prevented CAF activation through the suppression of α-SMA, MMP-9, TGF-β1, and IL-6. IL-6 is primarily expressed by CAFs and promotes cancer stemness. Clinical data revealed that IL-6 was prominently expressed in the stromal portion of GC tissues, and IL-6 upregulation in GC tissues was correlated with poor responsiveness to chemotherapy. CAF-mediated inhibition of chemotherapy-induced apoptosis could be abrogated by the anti-IL-6 receptor monoclonal antibody [47,48]. This study provided evidence for crosstalk between gastric cancer cells and CAFs by IL-6, which is a key contributor to chemoresistance. 

Cells of five-Fu are a common first-line chemotherapeutic drug for the treatment of gastric cancer. ER stress confers 5-FU resistance in breast cancer, colon cell, and hepatocellular carcinoma through the GRP78-mediated pathway [26,27,49]. Our data demonstrated that 5-FU induced GRP78-mediated gastric cancer stemness and that the inhibition of GRP78-mediated stemness by ISL may enhance chemosensitivity and suppress tumor growth in a xenograft animal study. 

A positive association between GRP78 expression and unfavorable overall survival was found in patients with gastric cancer. Zheng et al. demonstrated that GRP78 mRNA expression was higher in gastric cancer than normal tissues by performing bioinformatics analysis. Furthermore, a higher GRP78 mRNA expression was detectable both in intestinal-type carcinoma and diffuse-type counterpart in The Cancer Genome Atlas (TCGA) dataset [50]. GRP78 inhibition may possess potential benefits in clinical gastric cancer therapy.

## 5. Conclusions

Regarding cancer stemness and chemoresistance in gastric tumors, systemic chemotherapy with multiple drugs may be an effective strategy for patients with recurrent gastric cancer. Awareness regarding the importance of natural products for human health has been increasing. Dietary phytochemicals are candidates for anticancer research and can be crucial targets for cancer stemness. Natural products may be vital in the development of novel anticancer drugs. Our study demonstrated that ISL suppressed, not only GRP78-mediated gastric cancer stem cell–like characteristics, stemness-related proteins, and cancer-associated fibroblast activation in the tumor microenvironment, but also gastric tumor growth in xenograft animal studies. The results of this study indicate ISL as a promising candidate for clinical use in combination chemotherapy.

## Figures and Tables

**Table 1:** Clinicopathological characteristics of GC patients.

Characteristics	No. Pf Patients
Normal tissue	20
Gastric carcinoma	20
**Gender**	
M	10
F	10
**Age**	
≥60	13
<60	7
**Histologic grade**	
Moderately differentiated	9
Poorly differentiated	11
**TNM stage**	
I	5
II	5
III	5
IV	5
**LN metastasis**	
Absence	6
Presence	14
**Survival (TNM stage)**	
I	5/5
II	2/5
III	1/5
IV	0/5

Histomorphology of all tumor specimens was confirmed with H&E staining according to the International Union against Cancer TNM classification; M, male; F, female; LN, lymph nodes.

## Data Availability

All data sets generated or analyzed in this study were included in the published article. Detailed data sets supporting the current study are available from the corresponding author upon request. This study did not generate new codes.
